# Relationship Between Hemoglobin and Blood Pressure Levels in the Context of Chronic Morbidity Among Older Adults Residing in a Developing Country: A Community-Level Comparative Cross-Sectional Study

**DOI:** 10.7759/cureus.19540

**Published:** 2021-11-13

**Authors:** Tandra Ghosh, Tanveer Rehman, Farhad Ahamed

**Affiliations:** 1 Physiology, All India Institute of Medical Sciences, Kalyani, Kalyani, IND; 2 Community Medicine and Family Medicine, All India Institute of Medical Sciences, Kalyani, Kalyani, IND

**Keywords:** anemia, older adults, chronic diseases, hemoglobin, blood pressure

## Abstract

Objectives: The objective of this study is to assess the association between hemoglobin (Hb) and blood pressure (BP) levels among community-dwelling older adults.

Materials and Methods: This comparative cross-sectional study was conducted in April 2021 in West Bengal, India. Individuals who are receiving treatment for hypertension, or had a history of blood transfusion, or had a history of intake of iron and vitamins were excluded from the study. A total of 81 and 106 individuals were recruited in “Group 1” (no self-reported comorbidity) and “Group 2” (self-reported comorbidity present), respectively.

Results: The mean (SD) age of the participants (n = 187) was 67.4 (7.4) years; 54% (n = 101) were males; and 45.4% (n = 85) were obese. We found a significant association of Hb level with systolic blood pressure (SBP) (r = 0.22, p = 0.04) and mean arterial pressure (MAP) (r = 0.22, p = 0.04) in “Group 1”. The quantum of increases in SBP, diastolic blood pressure (DBP), pulse pressure (PP), and MAP with one gram/dl change in Hb level were 3.24 (95% CI: -0.75 to 7.24) mmHg, 1.17 (95% CI: -0.84 to 3.20) mmHg, 2.06 (95% CI: -0.67 to 4.81) mmHg, and 1.87 (95% CI: -0.65 to 4.39) mmHg, respectively, in “Group 1” after adjustment for age, gender, and body mass index. The findings were inconsistent in “Group 2”, and the changes in Hb level were 0.5 (95% CI: -3.77 to 2.77) mmHg, 0.92 (95% CI: -0.72 to 2.75) mmHg, -1.42 (95% CI: -4.09 to 1.24) mmHg, and 0.45 (95% CI: -1.05 to 2.40) mmHg, respectively.

Conclusion: Hb level has a positive correlation with SBP and MAP only in those without comorbidities.

## Introduction

The world is experiencing rapid growth in the older adult (aged ≥ 60 years) population. Older adults acquire multiple functional limitations due to the accumulation of degenerative changes with aging. This mandates the quality of life (QoL) to be considered as a better indicator of health than the overall increase in life expectancy among older adults [1]. Blood hemoglobin (Hb) level and blood pressure (BP) are two important modifiable parameters that have been reported to affect QoL of older adults, often independent of other factors.

A large number of older adults suffer from high blood pressure (BP) (hypertension), which contributes to significant mortality and morbidity [2,3]. Anemia, a major nutritional deficiency disorder among older adults, is independently associated with decreased functional ability, increased risk of dementia, increased risk of falls, and increased adverse cardiovascular and neurological events [4]. A study done in China has found a positive correlation between BP and Hb levels [5]. However, the evidence is limited in developing countries where the prevalence of anemia and hypertension is considerably high among older adults and often co-exist in this age group [6,7]. Thus, we wanted to study the relationship between Hb and BP in older adults to understand how does the Hb level increase the cardiovascular risk in terms of an increase in BP.

Older adults generally acquire multiple other chronic diseases with the advancement of age [8]. The presence of chronic diseases increases inflammatory cytokines in the body which in turn affects the absorption of key nutrients such as iron required for the synthesis of hemoglobin and also increases the BP via various mechanisms [9,10]. So, we further decided to examine the relationship between Hb level and BP in older adults after stratifying them according to the presence of chronic diseases. In this study, we have assessed the correlation between Hb level and BP along with the quantum of change in BP with a unit change in Hb level among the community-dwelling elderly population after stratifying them according to the presence of chronic diseases.

## Materials and methods

Study design and setting

A comparative cross-sectional study was conducted during April 2021 in the urban field practice area of a medical college in Kalyani. Kalyani is one of the earliest planned cities of India and is located around 50 kilometers away from the metropolitan city of Kolkata, West Bengal. The municipality area is divided into 21 ward/block areas - all similar in sociodemographic and economic characteristics and caters to a population of around one lakh.

Study participants

All older adults (aged ≥ 60 years) residing in the ward/block areas of Kalyani municipality for at least the past one year were eligible to participate in the study. Individuals with a present history of receiving an anti-hypertensive group of medication, history of blood transfusion in the last six months, and history of intake of iron and folic acid and/or vitamin B12 in the last six months were excluded from the study as these parameters can influence the primary outcome variables.

Sample size and sampling method

A list of households was obtained from Kalyani municipality. There was a total of 22,100 households/families (in a standalone house or residential complex or flats) in the study area, and all were having a unique number provided by the municipality. A total of 1,112 households were listed, which had at least one eligible participant, and this had served as the sampling frame. Study households were selected from the sampling frame through simple random sampling using a computer-generated sequence. Assuming a correlation coefficient (r) of 0.27 [5], 80% power, 5% alpha error, and 10% non-response rate, we calculated a required sample size of 110 in each group. We decided to recruit 110 individuals with “no self-reported chronic morbidity” and 110 individuals with “self-reported chronic morbidity” from the randomly selected households (considering a 10% non-response rate). The operational definition for self-reported chronic morbidity was "anyone with a disease for which he/she was consuming medication for last six months".

Study tool

An interview was conducted using a structured questionnaire; the first part was used to collect information regarding demography and comorbidities. Individuals taking medicines for the last six months for conditions like diabetes, body or back pain, chronic obstructive pulmonary disease (COPD), osteoarthritis, or hypothyroidism were considered to suffer from chronic disease conditions. The second part comprised Hb estimation, anthropometry, and BP measurements.

Data collection

Data were collected using trained medical social workers (MSWs) under the supervision of resident doctors of the Department of Community Medicine and Family Medicine. The initial screening and data collection from the recruited individuals were done in house-to-house visits using the standard infection control precautions, and if any house was locked, then the next eligible household was approached. Initially, the age eligibility of the individual was assessed. If the individual was found to be eligible, she/he was asked “Do you have any disease for which you are taking medicine for the last six months?” If the answer was “No”, she/he was included in “Individuals with no reported chronic morbidity” (Group 1). If the answer was “Yes”, the exclusion criteria were applied and they were identified as “Individuals with reported chronic morbidity” (Group 2). The participants were contacted only once for the study, and data collection happened three times a week. Only one individual from each household was selected for the study. If any house had more than one eligible participant, then one of them was selected using the lottery method.

Anthropometry measurements such as weight and half-arm span were taken with height being estimated from arm span for calculating body mass index (BMI) [11]. Hb level was estimated by digital hemoglobinometer - HemoCue 301 (HemoCue AB-hemoglobin photometer, Ängelholm, Sweden) [12]. The cuvette was filled with capillary blood by the trained MSWs, and measurements were not deferred beyond 40 seconds [13]. BP was measured using a calibrated digital BP monitoring machine (Omron Healthcare Omron HEM-7156, Omron Healthcare Manufacturing Vietnam Co. Ltd., Japan). It was taken in a non-dominant hand after sitting for 15 minutes in a comfortable position, either in bed or chair [14]. The cuffed arm was held up by the observer or rested at heart level. Three readings were taken one minute apart; the first was discarded, and the mean of the second and third readings was used. All were recorded at the same time of the day, i.e., during afternoon hours.

Pulse pressure (PP) and mean arterial pressure (MAP) were calculated from systolic blood pressure (SBP) and diastolic blood pressure (DBP) using the standard formula [15].

Ethical approval

Ethical approval was obtained from Institute Ethics Committee (IEC) (Reference number: T/IM-NF/Kalyani/20/09). Informed consent was taken before the questionnaire was administered to the study participants.

Statistical analysis

Data were collected using EpiCollect version 5.0 and analyzed using Stata version 14 (Stata Corp, College Station, TX, USA). Descriptive statistics were presented as mean and standard deviation (SD) for continuous variables and frequencies with proportions for categorical variables. Pearson’s correlation coefficient (r) was used to measure the association between Hb level (considering it as exposure) and SBP, DBP, PP, and MAP (considering these as outcome variables), and the strength of correlation was graded accordingly [16]. The quantum of change in the outcome variable was estimated from the slope (β) in a simple linear regression model and expressed with a 95% confidence interval (CI). P value < 0.05 was considered statistically significant.

## Results

An initial screening was done among 220 individuals, out of whom 33 individuals refused to participate citing prior commitments. Finally, a total of 81 individuals were recruited in “Group 1”, i.e., with no self-reported comorbidity, and 106 were recruited in “Group 2”, which comprised of individuals taking treatment for diabetes (33.0%, n = 35), hypothyroidism (17.9%, n = 19), osteoarthritis (16.0%, n = 17), backache/chronic pain (15.1%, n = 16), and COPD (10.4%, n = 11) with multiple conditions coexisting.

**Figure 1:**
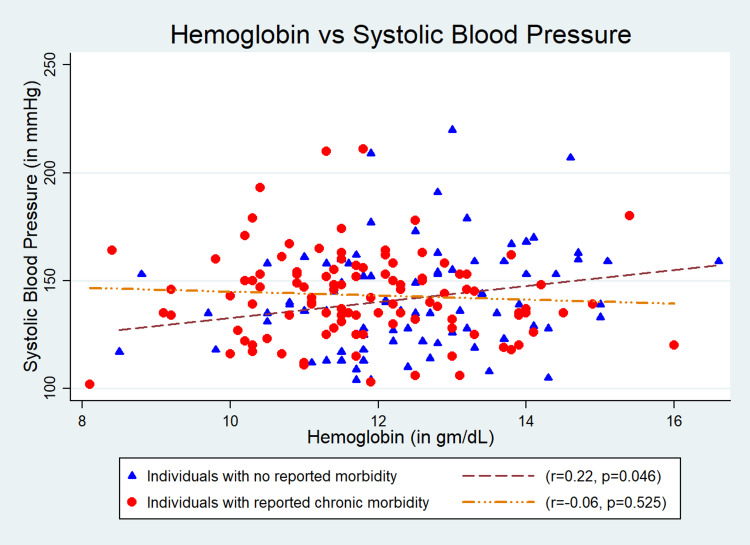
Correlation between hemoglobin and systolic blood pressure.

**Figure 2:**
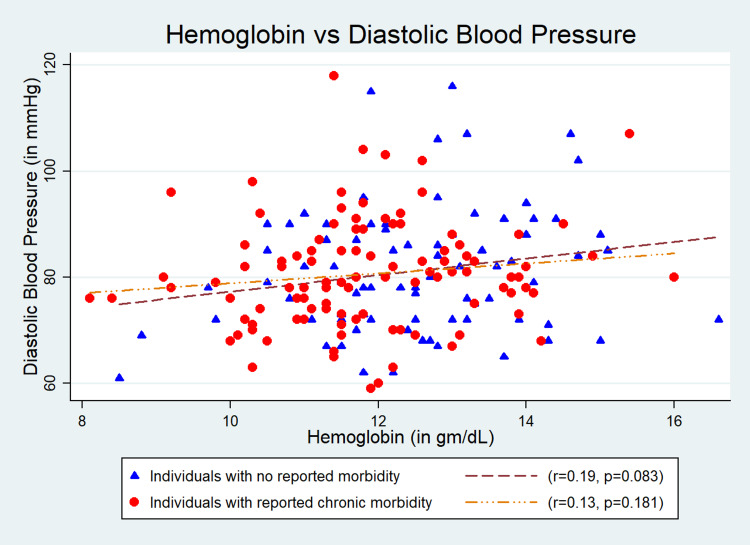
Correlation between hemoglobin and diastolic blood pressure.

**Figure 3:**
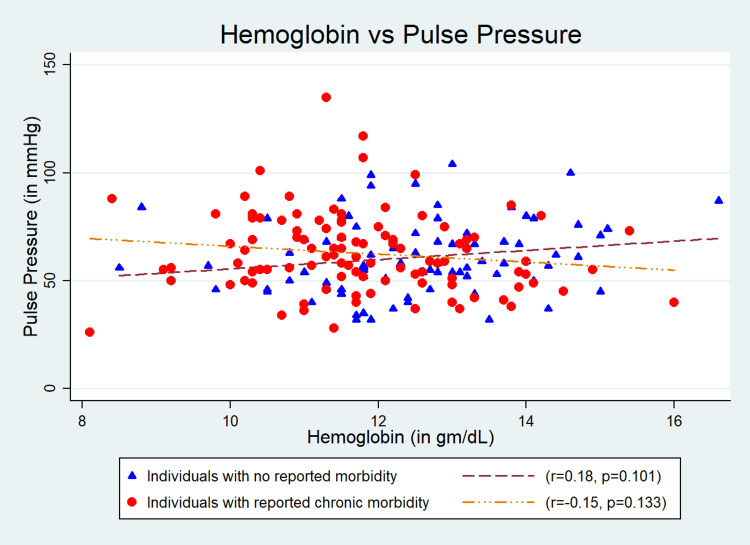
Correlation between hemoglobin and pulse pressure.

**Figure 4:**
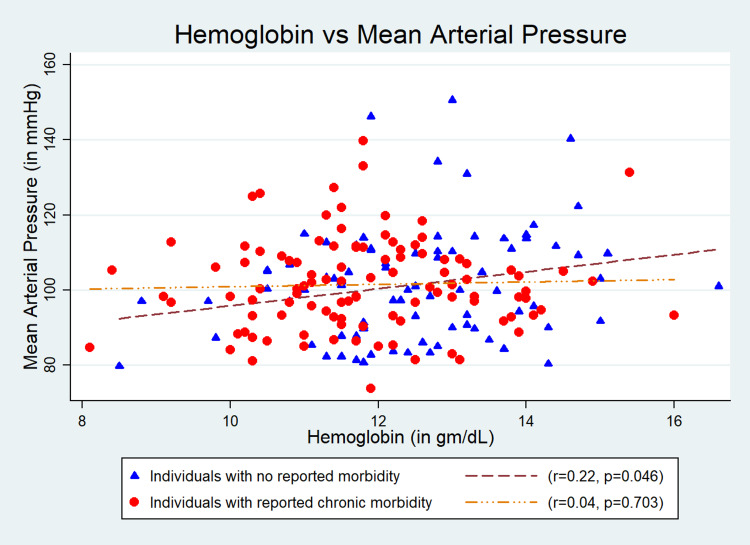
Correlation between hemoglobin and mean arterial pressure.

The mean (SD) age of the participants was 67.4 (7.4) years, and 54% (n = 101) of the study participants were males. Of total, 45.4% (n = 85) were obese (Table 1). The mean (SD) Hb level estimated was 12.5 (1.5) gm/dL in “Group 1” and 11.8 (1.4) gm/dL in “Group 2”. The mean (SD) SBP and DBP were 142.8 (21.2) and 80.6 (9.5) mmHg, respectively, for “Group 1” and 142.6 (23.6) and 81.0 (11.9) mmHg, respectively, for “Group 2”. Its association with SBP (r = 0.22, p = 0.04) (Figure 1) and MAP (r = 0.22, p = 0.04) (Figure 4) were statistically significant but was weakly correlated in “Group 1”. However, the association of Hb with SBP, DBP, PP, and MAP was not statistically significant in “Group 2” (Figures 1-4). The quantum of increases in SBP, DBP, PP, and MAP with one gram/dL change in Hb level in “Group 1” was 3.24 (95% CI: -0.75 to 7.24) mmHg, 1.17 (95% CI: -0.84 to 3.20) mmHg, 2.06 (95% CI: -0.67 to 4.81) mmHg, and 1.87 (95% CI: -0.65 to 4.39) mmHg, respectively, after adjustment for age, gender, and BMI. However, all these values were statistically not significant (Table 2).

**Table 1:** Demographic and morbidity profile of study participants without self-reported chronic morbidity (Group 1) and with self-reported chronic morbidity (Group 2) among the community-dwelling elderly population in West Bengal, India, 2021. *Column percentage, †Asia-Pacific classification.

Characteristics	Group 1 n (%)*	Group 2 n (%)*	Total n (%)*
Total	81	106	187
Age category (in years)	68.9 (8.0)	66.3 (6.8)	67.4 (7.4)
Youngest old (60-74 years)	77 (95.1)	80 (75.5)	157 (84.0)
Old (75-84 years)	4 (4.9)	19 (17.9)	23 (12.3)
Oldest-old (>85 years)	0 (0.0)	7 (6.6)	7 (3.7)
Gender			
Male	49 (60.5)	52 (49.1)	101 (54.0)
Female	32 (39.5)	54 (50.9)	86 (46.0)
Body mass index category^†^ (in kg/m^2^)			
Underweight (<18.50)	3 (3.7)	9 (8.5)	12 (6.4)
Normal (18.50–22.99)	25 (30.9)	28 (26.4)	53 (28.3)
Overweight (23.00–24.99)	9 (11.1)	28 (26.4)	38 (20.3)
Obesity (≥25.00)	44 (54.3)	41 (38.7)	84 (44.9)

**Table 2:** Quantum of change in blood pressure (mmHg) with one unit change in hemoglobin (gm/dL) level among the community-dwelling elderly study participants without self-reported chronic morbidity (Group 1) and with self-reported chronic morbidity (Group 2) in West Bengal, India, 2021. Dependent variables are systolic blood pressure (SBP), diastolic blood pressure (DBP), pulse pressure (PP), and mean arterial pressure (MAP); the independent variable is hemoglobin (Hb) level. The model was adjusted for age, sex, and body mass index. *The changes in SBP (p = 0.36), DBP (p = 0.11), PP (p = 0.88), and MAP (p = 0.14) across groups were insignificant.

Groups	SBP*			DBP*			PP*			MAP*		
	β	95% CI	P-value	β	95% CI	P-value	β	95% CI	P-value	β	95% CI	P-value
1	3.24	-0.75-7.24	0.11	1.17	-0.84-3.20	0.25	2.06	-0.67-4.81	0.14	1.87	-0.65-4.39	0.15
2	-0.50	-3.77-2.77	0.76	0.92	-0.72-2.57	0.27	-1.42	-4.09-1.24	0.29	0.45	-1.50-2.40	0.65

## Discussion

In this study, we have investigated the association between Hb level and BP among community-dwelling older adults (aged ≥ 60 years). As comorbidities are common with aging, we also have separately compared the association between Hb and BP in individuals with no comorbidity (Group 1) and individuals with one or more than one comorbidity (Group 2). We have found a consistent trend of increase in SBP, DBP, PP, and MAP with an increase in Hb among individuals with no comorbidity (Group 1). However, the correlation was not consistent in Group 2. Previously done two studies in China and Netherlands reported a similar positive correlation of Hb with SBP and DBP [5,17]. Physiologically, BP increases with age due to arteriosclerotic structural alterations and calcification of blood vessels. Nonetheless, multiple Hb-mediated mechanisms have also been reported to cause increased arterial stiffness. It has been found that a high Hb level causes damage to the vascular endothelium and results in increased arterial stiffness [18]. Increased Hb level had also been found to increase peripheral vascular resistance by altering blood viscosity as well as causing vasoconstriction by quenching the nitric oxide (NO) of the endothelium. Both the mechanisms result in increased BP [9,19,20]. In addition, specific genetic predispositions like an increased expression of endothelin 1 induced by increased Hb level had also been reported to cause increased arterial stiffness, which has a definite role in rising BP [21]. High Hb level has been reported as an independent determinant of soluble CD40/CD40L level in the blood, which can induce endothelial damage [22]. Accumulation of endothelial damage increases peripheral vascular resistance and DBP.

People with chronic morbidities usually manifest with a high level of circulatory inflammatory cytokines like tumor necrosis factor-α (TNF-α) and interleukin-6 (IL-6). Both of these have a direct inhibitory action on iron absorption from the gut and the release of iron from store cells via the hepcidin-mediated pathway, thereby causing anemia [9]. As a result, the relationship between Hb and BP becomes more complex in the presence of chronic inflammatory conditions. This was the probable reason behind the observed inconsistent relationship between Hb and BP in Group 2 of our study, and this needs to be studied further with larger sample size. Moreover, any of the previously done studies did not differentially assess the association of Hb with BP in individuals with or without comorbidities. So, we could not compare our results with any available observation.

The quantum of increase in SBP and PP with per gram/dL increase of Hb was 3.24 mmHg and 2.06 mmHg among individuals in Group 1 after adjusting for age, gender, and BMI. The observation was in consonance with an earlier study [17]. It has been estimated that an increase of 20 mmHg in SBP significantly increases adverse cardiovascular events [19]. We found a rise of about 3 mmHg of SBP with a 1 gm/dL increase in Hb across the study population. This indicates that treatment of anemia in older adults especially in the presence of hypertension needs to be monitored with extra precaution as an increase in Hb may further aggravate or add up to the existing cardiovascular risks. There are reports that a slightly low Hb level is beneficially associated with arterial stiffness in elderly females, thereby reducing the risk of adverse cardiovascular incidents [23]. The observed increase in PP with a change in Hb level can also be considered an important concern. A high level of PP (>80 mmHg) is strongly associated with arterial stiffness and can act as an independent risk factor of cardiovascular complications [24]. This again suggests the necessity of close monitoring of anemia treatment in this age group.

This was a comparative cross-sectional study, so temporality cannot be established. The chance of random dilution bias could not be canceled as BP was measured in a single visit using a digital sphygmomanometer. We grouped the individuals based on self-reporting of chronic morbidity. Assessment of serum inflammatory bio-marker would have confirmed the disease activity and further strengthened the relationship. No earlier study from developing nations differentially assessed the correlation of Hb with SBP, DBP, PP, and MAP among individuals with or without comorbidity. Our study findings provide this important information.

## Conclusions

Hb level has a positive correlation with SBP and MAP only among the older adults without apparent comorbidity. Further research is required to assess the above relationship among the people with comorbidities as the prevalence of anemia and hypertension is high in older adults in the developing countries.
